# Object Feature Memory Is Distorted by Category Structure

**DOI:** 10.1162/opmi_a_00170

**Published:** 2024-11-22

**Authors:** Marlie C. Tandoc, Cody V. Dong, Anna C. Schapiro

**Affiliations:** Department of Psychology, University of Pennsylvania, Philadelphia, PA, USA; Department of Psychology, Princeton University, Princeton, NJ, USA

**Keywords:** category learning, memory distortion, neural network model

## Abstract

Memory systems constantly confront the challenge of capturing both the shared features that connect experiences together and the unique features that distinguish them. Across two experiments, we leveraged a color memory distortion paradigm to investigate how we handle this representational tension when learning new information. Over a thirty-minute period, participants learned shared and unique features of categories of novel objects, where each feature was assigned a particular color. While participants did not differ in how accurately they remembered these features overall, when inaccurate, participants misremembered the color of shared (relative to unique) features as more similar to the category’s average color, suggesting more integration of shared features in memory. This same rapid representational warping manifested in a neural network model trained on the same categories. The work reveals how memories for different features are rapidly and differentially warped as a function of their roles in a category.

## INTRODUCTION

Information varies in how reliably it is observed across experiences. There are general patterns that link experiences together (we observe varied species of birds flying) and at the same time we notice idiosyncratic details that distinguish them (flamingos but not sparrows have long legs). How do we build memory representations that effectively capture both kinds of information? This presents a challenge for our memory systems as these forms of knowledge are best supported by different representational formats: Learning shared features benefits from allowing information to overlap across instances to capture similarities, while learning unique features benefits from keeping instances separate to reduce interference.

This tension in representing general and specific information has led to an abundance of research into how the brain can accomplish this feat (Bowman et al., 2020; Brunec et al., 2020; Frank et al., 2019; McClelland et al., 1995; Richards & Frankland, 2017; Schapiro, Turk-Browne, et al., 2017; Xu & Südhof, 2013). One solution is that we could prioritize one kind of information in memory over the other, such as by forgetting unique features in order to generalize (Richards & Frankland, 2017). Another solution is to keep track of both by representing information differently depending on its type. Several studies have shown that the brain has the capacity to employ integrated and separated memory representations in tandem (Bowman et al., 2020; Brunec et al., 2020; Fernandez et al., 2023; Molitor et al., 2021; Samborska et al., 2022; Schapiro, Turk-Browne, et al., 2017; Schlichting et al., 2015). While this suggests that it is possible for us to simultaneously represent information in a general or specific way, it is unclear whether shared and unique information themselves differentially make use of these representational signatures. We explore these ideas by probing the memory representations of different features as humans and models learn novel object categories.

In particular, we aim to understand how shared and unique features are represented according to their different computational needs, with the prediction that shared features will form more integrated representations relative to unique ones. This prediction is motivated by findings that items that reliably co-occur in similar temporal, relational, and spatial contexts, or that are members of the same category, quickly come to be represented similarly, especially in the hippocampus (Brunec et al., 2020). But this work on item co-occurrence does not tell us how different kinds of features within an individual item might be represented, structure which we know to play an important role in learning (Solomon & Schapiro, 2024).

To evaluate the representations of shared versus unique features, we measure distortions in memory for these features using color. Color memory distortion paradigms have proven to be effective behavioral assays for item representations in both working memory (Chunharas et al., 2022; Zhang & Luck, 2008) and long-term memory (Brady et al., 2018; Chanales et al., 2021; Miner et al., 2020; Saito et al., 2023). Many of these studies report attraction biases, where participants misremember an item’s color as more similar to the color of other studied items. For example, after viewing multiple colored objects of the same category (e.g., airplanes), color memory for an object is distorted towards the average color of its category (Brady et al., 2018). Attraction may emerge especially in tasks that promote integration of information in memory (Chanales et al., 2021). On the other hand, when a task requires separating information, repulsion has been observed, where the colors of competing items are remembered as less similar to one another (Chanales et al., 2021). Recent work has found that these color memory distortions map onto neural representational similarity measures (Zhao et al., 2021). Color memory thus provides a behavioral tool to quantify how memory representations might shift as a function of experience. Thus far, these approaches have been applied to item-level information. We extend this approach by leveraging color memory distortions to probe whether features within an item are differentially shaped by their role in a category.

**Figure 1.**
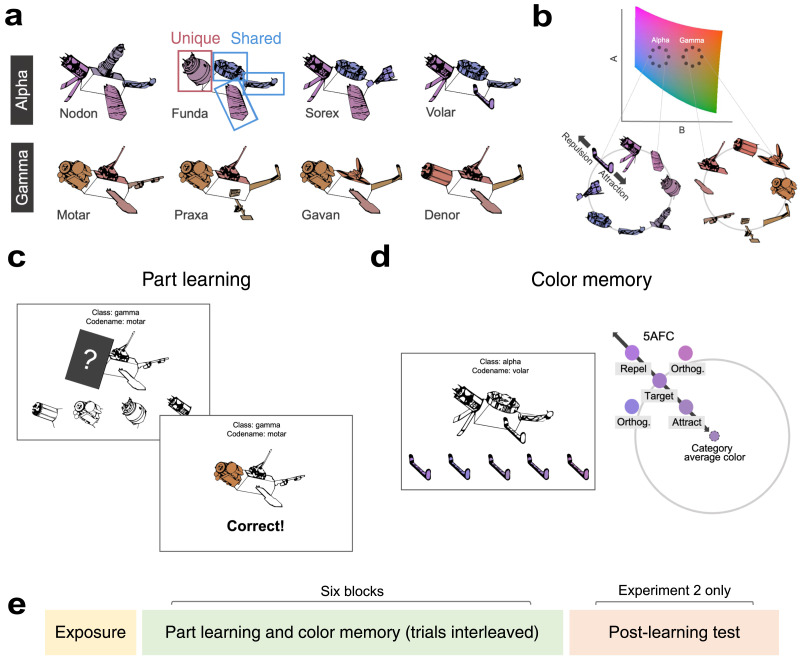
**Stimuli and task.** **(a)** Participants learned eight novel satellite objects from two categories (Alpha, Gamma). Each satellite had a unique codename (i.e., Nodon, Funda, etc.) and was made up of four parts. Satellites within a category shared most of their parts but each had one unique part. **(b)** The color of the parts for each category were arranged in a circle in a slice of 2D continuous color space. Color memory could then be measured as attraction towards the category center or repulsion away from it. **(c)** On part learning trials, a satellite was shown alongside its class and codename with one part covered. Participants selected the missing part from four options, repeating the trial until they chose correctly. Once correct, participants viewed the correct part on the satellite with its color. **(d)** On color memory trials, participants chose a part’s color in a 5AFC. Options included the target, a foil closer to the category’s average color, a foil farther away from the category’s average color, and two foils orthogonal to the attract-repel line. No feedback was given on these trials. **(e)** The main phase of the experiment proceeded in six blocks where participants alternated between part learning and color memory trials. In Experiment 2, a post-learning test was added which also assessed part and color judgments for novel satellites.

We trained human participants (Experiment 1: *n* = 85, Experiment 2: *n* = 109) on two novel categories of cartoon satellite objects (Schapiro, McDevitt, et al., 2017; Schapiro et al., 2018; Figure 1a). Each satellite had three parts shared with other satellites from the same category and one part specific to that particular satellite. Each part was assigned a color drawn from a 2D slice of CIELAB color space, with colors from the same category arranged in a circle (Figure 1b). Participants learned the parts via a feature inference task (Figure 1c), and color memory (Figure 1d) was continuously tested throughout six blocks of learning (Figure 1e). Across two experiments, we found that shared feature memory was distorted toward the category center while unique features resisted this attraction bias. The attraction for shared features persisted even when tested as part of a novel exemplar. We simulated the task in our neural network model of the hippocampus (C-HORSE; Complementary Hippocampal Operations for Representing Statistics and Episodes; Schapiro, Turk-Browne, et al., 2017), finding that shared features were represented more similarly to same-category features than were unique features, mirroring the human data. Together, the results demonstrate how feature representations rapidly warp to reflect the structure of the environment.

## METHODS

### Experiment 1

#### Participants.

85 participants (41 females, 39 males, mean age = 19.82, range = 17–26) from the University of Pennsylvania participated online in exchange for course credit. Demographic data from three participants were not collected due to technical difficulties. Data from 9 additional participants were excluded because they did not achieve above-chance performance in the last block of part learning. The study protocol was approved by the local Institutional Review Board.

#### Stimuli.

Line drawings of eight cartoon satellite objects were used as stimuli (Schapiro, McDevitt, et al., 2017; Schapiro et al., 2018), divided into two categories. Each satellite had a class name (Alpha or Gamma), a unique codename (Gavan, Sorex, etc.), and four visual parts. Each satellite had three parts that were shared with other satellites from the same category (shared features) and one part that was unique to that particular satellite (unique features). There were thus eight parts to learn per category (16 parts total). Parts were never shared across categories. The assignment of parts, codenames, and class names was randomized for each participant.

For each participant, a color was uniquely assigned to each of the 16 parts. Part colors were drawn from a 2D slice of CIELAB color space with a fixed lightness value of 60. The same slice was used for all participants. Prior work has successfully used 2D slices of CIELAB to assess systematic memory distortions (Miner et al., 2020). We selected the 16 colors by drawing two circles in this 2D slice (radius: 14, circle 1 center: A = 22, B = −25; circle 2 center: A = 22, B = 25) such that the color coordinates for the eight parts from the same category were arranged in a circle (Figure 1b). Perceptually this resulted in one category’s parts being more blue and purple and the other category being more brown and orange. Distance between adjacent colors on a circle was 10.71 CIELAB units. We used this circular arrangement because there is an easily interpretable average color for each category, which is the center of the circle, allowing us to assess memory distortions towards (attraction) or away from (repulsion) this center. The same color coordinates were used for all participants. This meant that participants all saw the same colors, but the assignment of the colors to parts was pseudorandomized for each participant as follows. Within the circular arrangement described above, we created two part-to-color mappings that were randomly assigned to participants (Supplementary Figure 1). The only difference between these two arrangements was that the possible color coordinates for shared and unique features were swapped. Parts were then randomly assigned to one of the color coordinates for each participant constrained by the condition assignment. Both these assignments had the constraint that two parts of the same type (head, tail, back, leg) from the same category were always two positions away on the circle.

#### Procedure.

Before starting the experiment, participants were told that they would take on the role of an engineer who would encounter satellites built by two different labs (Alpha Lab and Gamma Lab), each with a unique codename (Gavan, Sorex etc.). They were told that their job was to learn both the parts and the colors of the parts, and that satellites built from the same lab tended to share parts. After these instructions, participants viewed all uncolored satellites on the screen together for 5 seconds.

Before the main phase of the experiment (Figure 1e), participants completed a short initial exposure phase (mean duration = 3.8 minutes) to familiarize them with all eight satellites and their parts. On each trial, an uncolored satellite was displayed beneath its class and code name. A box highlighted each visual part of the satellite in a random sequence to encourage participants to attend to each part. When a box appeared, participants were instructed to click the indicated part. After clicking the part, participants saw the part’s color for 3 seconds before the next part was highlighted. After all four parts of a satellite were clicked, participants were asked to recall the class and code names by clicking the correct name in an alternative forced choice (for codenames all eight options were shown).

The main phase of the experiment then proceeded in 6 blocks (mean duration = 27.6 minutes). Each block consisted of 16 part learning trials (part trials) and 16 color memory test trials (color trials). Within a block, each of the 16 parts was queried on part trials and color trials. To obtain a continuous assessment of color memory as part learning progressed, these trial types alternated. Color trials always followed part trials, but the part tested on the color trial was not the part tested on the preceding color trial, or the two part trials before that. Trial order was otherwise shuffled randomly. A 1.5 s interval separated the trials. During this time participants were told the class and codename of the upcoming satellite and whether the upcoming trial was a part trial (indicated by a hammer icon and the word “build”) or color trial (indicated by a paintbrush icon and the word “paint”). Part trials were self-paced.

Shared features were queried on part trials and color trials across all of the three satellites with that shared feature. We chose one satellite to query each shared feature for each block. The satellite chosen in a block was pseudorandomized such that by the end of learning, all satellites were seen an equal number of times. On each part trial, an uncolored satellite (along with its class and codename) was presented with one part covered by an occluding box with a question mark (Figure 1c). A four alternative forced choice (AFC) test of parts was presented at the bottom of the screen (part location randomized). The text “Click the missing part” was always present on screen during the trial. After clicking an option, “Correct” or “Incorrect” would appear as feedback. If incorrect, participants would repeat the trial until selecting the correct part. After selecting the correct part, the occlusion box disappeared and participants saw the part on the satellite with its color for 4 seconds before moving on to the next trial. Because we carefully restricted the opportunities for participants to view a part’s color, and parts were queried an equal number of times, the frequency with which the colors of shared and unique features were seen was exactly matched during this learning phase. This design ensures that any differences in color memory for shared and unique features cannot be attributed to differences in frequency of color exposure. Showing participants only one part’s color at a time also avoids any perceptual interactions between multiple simultaneously presented colors. We constrained the part trial order such that the same satellite could not appear back to back, nor could a query on the same part type.

On each color memory test trial, an uncolored satellite was presented (along with its class and codename) with a rectangle surrounding one of its parts (Figure 1d). Five options for the part’s color were presented at the bottom of the screen (order randomized): the part’s true color (target) and four foils. Every trial had the same four foil types: An attract foil, a repel foil, and two orthogonal foils (Figure 1d). For each of the 16 parts, the foils were generated by drawing a line from that part’s color coordinate to its category circle center. A second line was created perpendicular to this line, intersecting with the part’s color coordinate. In each of the four directions surrounding the target, the foil color coordinates were drawn 5.35 LAB units away (exactly half of the distance between adjacent parts). Attract foils were closer to the category center than the true color and repel foils were further away. The same four foils were used each time a particular part’s color was tested. Color trials that timed out (at 15 seconds) during learning were excluded from analysis (a total of 42 trials across all participants). Although continuous responses are often used to test color memory distortions (e.g., responding on a color wheel), AFCs have been demonstrated to be sensitive to such distortions as well (Chunharas et al., 2022; Schurgin et al., 2020). Although it is possible that participants could infer the target by averaging all the options on screen, this would not impact shared and unique features differently, our contrast of interest.

### Experiment 2

#### Participants.

The effect size in Experiment 1 for the difference in color attraction for shared versus unique features in the last block was Cohen’s *d* = 0.24. A power analysis indicated that 109 participants would provide us with 80% power to detect the same effect. 109 participants (post-exclusions) were thus recruited online via Amazon’s Mechanical Turk (43 females, 66 males, mean age = 39.66, range = 21–63) in exchange for monetary compensation. Using the same performance-based criteria as the prior experiment, 15 additional participants were excluded. One participant was excluded due to being color blind. Seven participants were excluded because they failed to respond on greater than 10% of the trials in the test phase (trial timed out after 15 seconds on more than 10% of trials). A participant who was 71 years old (more than 3 SD above the mean age in this experiment) was also excluded. Finally, we excluded 2 participants displaying behavior highly suspicious of cheating (performance of 100% in Block 1).

#### Stimuli.

Satellite stimuli were identical to Experiment 1. For the post-learning test, 8 novel satellites were created to evaluate whether color memory distortions persisted in the context of unstudied satellites. These satellites belonged to one of the two classes, but had one novel part (for example a new “tail”).

#### Procedure.

An identical procedure was used for the replication in Experiment 2 except for the following changes: (1) a shortened initial exposure phase (mean duration now 2.7 minutes), and (2) a minor modification to how satellites were assigned to color memory trials: In Experiment 1 the satellite used to query a shared part on part trials was always the same satellite used to query that same shared part on color trials within a block (e.g., in Block 1 the *Alpha* category’s shared satellite “head” was queried on *Nodon* on both part and color trials). In Experiment 2 this constraint was lifted to allow for more variability, such that it was possible that a different satellite could be used to query that shared feature on part and color memory trials within a block (e.g., in Block 1 the *Alpha* category’s shared satellite “head” could be queried on *Nodon* for the part trial but on *Sorex* for the color trial). Shared and unique part colors were seen with equal frequency in the learning phase, but in this version we corrected a difference in frequency in the short initial exposure phase. Each of the eight exemplars was still shown one at a time, but now feature colors were only displayed for one unique and one shared feature for each satellite. This meant that the colors of shared and unique features were now seen with equal frequency during initial exposure. During the main learning phase, 47 color trials were excluded for timing out, across all participants.

#### Post-Learning Test.

The test phase consisted of a total of 72 part trials and 72 color trials. This phase was similar to the main phase of the experiment except (1) no feedback was given (i.e., participants were not told if the selected part was correct and did not see the part’s correct color) and (2) novel satellites were also queried on part and color trials. This meant that for both part and color trials, there were now three feature types that were queried: unique features on trained satellites, shared features on trained satellites, and shared features on novel satellites. Novel satellites were always shown with their class name, but had no codename. 11 color memory test trials were excluded across participants because the 15 second response window timed out.

### Mixed Effects Models

Mixed effects models included feature type (shared, unique) and block number (1–6) as interacting predictor variables. In all models, feature type (shared or unique) was contrast coded (shared = −1, unique = 1), block number was mean centered, and a random intercept for each subject was included. For part learning, a binomial generalized linear mixed effects model was used to predict at a trial level whether participants selected the correct part (correct = 1, incorrect = 0) on their first attempt on part trials. For color attraction bias, a linear mixed effects model was run to predict color memory biases. Each color trial response was coded as 1 = attract foil; 0 = target or orthogonal foil, −1 = repel foil. The model was then run to predict these coded responses as a function of feature type and block. For color accuracy, a binomial generalized linear mixed effects model was used to predict at a trial level whether participants selected the correct color (correct = 1, incorrect = 0) on color memory trials. Linear mixed effects models were run in R using the *lme4* package (Bates et al., 2014). The *lmerTest* package was used to obtain *p* values, and degrees of freedom were obtained using the Satterthwaite’s degrees of freedom method. Degrees of freedom are not reported for binomial generalized linear models (i.e., the mixed effects logistic regression models predicting binary outcomes for part learning and color accuracy) because degrees of freedom are less straightforward to calculate in the context of non-normal distributions.

### Neural Network Model Simulations

To simulate our paradigm, we employed C-HORSE, a neural network model of the hippocampus, that accounts for its roles in episodic memory, statistical learning, associative inference, and category learning (Heffernan et al., 2021; Schapiro, Turk-Browne, et al., 2017; Sučević & Schapiro, 2023; Zhou et al., 2023). To overview, the model has two main pathways, the trisynaptic pathway (TSP) and monosynaptic pathway (MSP), which have anatomical properties that lead to specialization in representing specifics and generalities, respectively. The model was trained via Contrastive Hebbian Learning to reproduce the satellite stimuli used in the human experiments, and activity throughout the network was recorded over the course of this training in order to study the model’s behavior and learned internal representations.

Model simulations were conducted in the Emergent environment (Aisa et al., 2008), and files for running the model can be found at https://github.com/schapirolab/hip-sl. The only changes from this version were to the input/output dimensionality (now 26) and a decrease in inhibition in layer DG (now Gi = 6) to allow activity to propagate smoothly under the new input dimensionality. Additional model description and details can be found in Schapiro, Turk-Browne, et al. (2017).

#### Model Architecture.

The model consists of an input layer and output layer representing the superficial (EC_in) and deep layers (EC_out) of entorhinal cortex and three hidden layers representing dentate gyrus (DG), cornu ammonis 3 (CA3), and cornu ammonis 1 (CA1) hippocampal subfields (Figure 5a). There are two main pathways connecting these layers: The MSP, which connects EC and CA1 directly, and the TSP, which connects EC_in to CA1 through DG and CA3.

Each layer is composed of units, which each have an activation value at each processing cycle ranging from 0 to 1. Unit activity is intended to simulate the firing rate of a neuron or small population of neurons. A unit’s activity is proportional to the activity of all units sending connections to that unit, weighted by connection weights between them. The pathways (arrows in Figure 5a) determine which layers have connected units. The key projections within the TSP are sparse, meaning that each unit in a sending layer has connections to a small proportion of units in the receiving layer (proportions: EC_in to DG = 0.25, EC_in to CA3 = 0.25, DG to CA3 = 0.05). The TSP also has high inhibition, which leads to few units being active at a time. The sparse connections and high inhibition in the TSP together drive pattern-separated representations in DG and CA3, where even very similar inputs coming through EC_in activate distinct sets of units in these areas. Projections into and out of CA1 have full connectivity, meaning that every sending unit connects to every receiving unit. Inhibition is also relatively lower in CA1. These properties lead to more overlapping representations in CA1. There is a separate Input layer (not shown in Figure 5a) with the same dimensions as EC_in where external input was clamped (units corresponding to a presented pattern set to have value 1 and others set to 0), with one-to-one connections into EC_in. This layer establishes external input for EC_in while also allowing for the influence of “big-loop” recurrent activity from EC_out to EC_in. EC_out to EC_in connectivity is also one-to-one.

#### Stimuli and Training.

Stimuli were constructed to mirror the experiments, with four satellites in each of two categories (see Supplementary Table 1). Each satellite feature (class name, code name, and parts) corresponded to one input unit. The Input, EC_in, and EC_out layers of the network were constructed as 26-unit vectors, appending the features for the two orthogonal categories. For every training trial, the model was presented with a full satellite pattern in the Input layer and was trained to reproduce that full pattern on EC_out. Connection weights throughout the network were adjusted to improve performance on this auto-encoding task using a form of Contrastive Hebbian Learning (Ketz et al., 2013; Schapiro, Turk-Browne, et al., 2017): Each trial had a “plus” phase, where the correct full satellite pattern was clamped on EC_out and activity was allowed to propagate through the network. Each trial also had two “minus” phases, where the TSP and MSP were allowed to propagate activity independently (with the other pathway turned off), in turn, with clamped input but not output. The patterns of activity for connected pairs of units were recorded in each phase, and connection weights between connected units were adjusted in proportion to the differences between these phases. The result of this process is that the patterns of coactivity between units in each of the minus phases become more similar to the patterns of coactivity in the plus phase. There were 16 trials in each training epoch, with every satellite presented twice (in random order). Each model was trained for six epochs, matching the six blocks of training/testing that humans underwent.

#### Testing.

Networks were tested before any training (epoch 0) and after every training epoch. We tested with full satellite patterns and individual satellite features as input (Supplementary Table 1). We recorded activations after 100 cycles of processing for test trials in which the connection between EC_out and EC_in was lesioned and test trials in which the connection was intact. Preventing activity from spreading from EC_out to EC_in allows us to assess the representations in CA1 before they have the potential to influence DG and CA3 through “big-loop” recurrence, which provides a clearer picture of the different contributions of the subfields. We refer to these tests where CA1 does not influence DG and CA3 as the “initial response.”

#### Lesions.

To assess the contributions of the MSP and TSP to shared/unique feature representations, we simulated lesions to these pathways. For models with the MSP lesioned (“TSP only”), we set the projection strength from EC_in to CA1 to 0. For TSP lesions (“MSP only”), we set the projection strengths from EC_in to DG and CA3, DG to CA3, and CA3 to CA1 to 0. Lesions were present during both training and testing.

#### Random Initialization.

Analyses were based on 50 model initializations where each initialization corresponds to randomized weights and randomized configuration of the sparse projections in the TSP. Results were averaged across networks.

## RESULTS

### Experiment 1

#### Part Learning.

**Figure 2.**
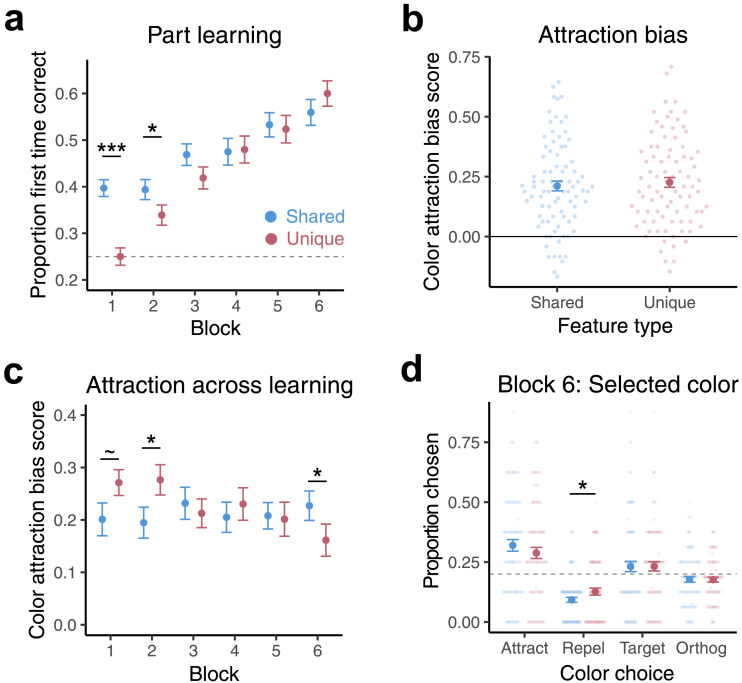
**Part learning and color memory in Experiment 1.** **(a)** The proportion of trials in which the correct satellite part was selected on part trials for shared (blue) and unique (red) features across training blocks. **(b)** Overall color attraction bias scores for shared and unique features. An above-zero score indicates a participant was more often selecting colors closer to the category center. **(c)** Color attraction bias scores for shared and unique features across blocks. **(d)** The proportion of color memory trials in which each color option (Attract, Repel, Target, Orthogonal) was selected for shared and unique features in the sixth block. Dashed-lines denote chance performance. Error bars denote ±1 standard error of the mean. Each small dot corresponds to a participant. Large dots depict group means. Significance stars reflect uncorrected *p* values. ***p < .001, *p < .05, ∼*p* < .1.

To examine satellite part performance over the course of learning, we ran a binomial generalized linear mixed effects model with feature type (shared, unique) and block (1–6) predicting accuracy (0, 1) on each part trial (Figure 1c). Participants showed strong evidence of learning across blocks (*B* = .22, *SE* = .01, *z* = 16.14, *p* < .0001; Figure 2a). Participants were overall better at inferring missing shared than unique parts (*B* = −.08, *SE* = .02, *z* = −3.56, *p* = .0004), but there was a significant interaction between block and feature type (*B* = .07, *SE* = .01, *z* = 5.12, *p* < .0001). Post-hoc *t* tests on accuracy scores within each block revealed that participants were better at inferring missing shared than unique features in the first block (Block 1: *p* < .0001, FDR corrected *p* < .0001) but that performance for shared and unique features became increasingly matched in the remaining blocks (Block 2: *p* = .04, FDR corrected *p* = .13; Block 3: *p* = .11; Block 4: *p* = .79; Block 5: *p* = .56; Block 6: *p* = .17). Part learning was far from ceiling even in the last block of learning, suggesting that the observed lack of difference was not simply due to lack of sensitivity to detect a difference near ceiling.

#### Color Memory.

We next examined whether color memory for satellite parts was distorted by the category structure. We calculated an *attraction bias score* for each participant based on which colors they selected on the 5AFC color memory trials (Figure 1d). We coded the response on each trial to indicate the direction of the memory bias (whether it was closer or further away from the category center; 1 = attract foil; −1 = repel foil; 0 = target or orthogonal foil). Color attraction bias scores were then calculated as the average of these values. A positive score indicates that a participant misremembered colors as more similar to the category’s average color (attraction) whereas a negative score indicates that a participant misremembered colors as more dissimilar (repulsion).

Color attraction bias scores were significantly positive (*t*(84) = 11.74, *p* < .0001), and there was no difference in attraction bias scores for shared and unique features when averaging across all blocks (*t*(84) = −0.89, *p* = 0.38; Figure 2b). We assessed how color memory for the two part types changed over the course of learning by fitting a linear mixed effects model with feature type and block predicting color attraction bias on each trial. There was a main effect of block such that attraction decreased across learning (*B* = −0.01, df = 8030, *t* = −2.25, *p* = .02). We found a significant interaction, however, between feature type and block (*B* = −.01, *SE* = .004, *t* = −3.26, *p* = .001, Figure 2c). A post-hoc comparison of these estimated slopes revealed that the color attraction bias for unique features (*B* = −.02, *SE* = .01, df = 8030, *t* = −3.90, *p* = .0001) but not shared features (*B* = .003, *SE* = .01, df = 8030, *t* = 0.71, *p* = .47) declined across learning. Post-hoc *t* tests on bias scores in each block revealed that in the last block of learning participants showed somewhat greater attraction bias for shared than for unique features (*t*(84) = 2.26, *p* = .03), though this effect is marginal after multiple comparisons correction (FDR corrected *p* = .08). Taken together, attraction (misremembering a feature’s color as having been more similar to the average category color) for shared features did not change with learning, whereas unique features increasingly resisted this attraction bias as learning progressed. Participants showed some evidence of more attraction for unique than shared features early on in learning (Block 1: *t*(84) = −1.84, *p* = .07, FDR corrected *p* = .14; Block 2: *t*(84) = −2.24, *p* = .03, FDR corrected *p* = .08; see discussion below). There was no difference between shared and unique attraction in blocks 3–5 (*p*’s > .41). There was no correlation between a participant’s overall part performance and color attraction biases for either feature type (*p*’s > .14). Differences in color memory for shared versus unique features are therefore likely not due to differences in how well shared versus unique parts were learned.

We also ran a mixed effects model including color arrangement assignment as an interaction term (there were two randomly assigned possible arrangements; see Methods and Supplementary Figure 1). The interaction between feature type and block held with color arrangement added to the model (*p* = .002). However, the model revealed an interaction between feature type and color arrangement (*p* < .001), suggesting that the difference in attraction for shared versus unique features was more evident for some colors than for others. The interaction between feature type and block also remained significant (*p* = .001) when including color (the 16 color coordinates) as a factor in the mixed effects model. In sum, while color bias may be observed more for some color assignments than others, the effects hold when controlling for color.

What led to this decline in attraction for unique features across learning? One possibility is that color memory for unique features grew more precise than shared features, while another possibility is that memory was distorted in a different way. To adjudicate between these possibilities, we first ran a binomial generalized linear mixed effects model with feature type and block predicting color accuracy (as opposed to color bias). This analysis revealed no difference in overall color accuracy between shared and unique features (*p* = .17). Color accuracy improved across time (*B* = .06, *SE* = .02, *z* = 3.88, *p* = .0001), with no evidence for a difference in improvement between shared and unique features (interaction *p* = .38). This matched improvement in color accuracy across feature types suggests that the decline in attraction bias for unique features is not due to participants remembering the unique features more accurately. We next directly examined which color options participants were selecting in the 5AFC (amongst the target color, attract foil, repel foil, and two orthogonal foils). Proportion of target choice corresponds closely to the accuracy analyses reported above (with the difference being that those were estimated in the context of a mixed model), but here we provide insight into the patterns across foil choices. In the last block of learning, participants chose the repel foils more often for unique than for shared features (*t*(84) = −2.27, *p* = .03; Figure 2d). Unique features, relative to shared features, were misremembered as more *dissimilar* from the category’s average color by the last block of learning. No other differences between shared and unique features were observed for other color option types (*p*’s > .19). Replicating the color accuracy model results, there was again no difference in how often the target color was selected for shared versus unique features (*p* = .98). Together, these findings demonstrate that the differences in color memory between shared and unique features in the last block was not due to more veridical memory for the unique feature colors, but instead a different kind of distortion.

We found that whether a satellite’s part was shared or unique had consequences for how these features were remembered. Participants showed persistent color attraction biases for shared features, whereas unique features became increasingly resistant to this bias as learning progressed. Towards the end of learning, unique features showed less attraction than shared features, driven by participants misremembering the color of unique features as more distinctive relative to the average color of that category. The findings suggest that unique feature representations become less integrated with the category over the course of learning. Because participants saw the colors of shared and unique features with equal frequency, this finding cannot be attributed to having seen the color of shared parts more often. This does not rule out the possibility that participants were imagining or retrieving the colors of shared and unique features with different frequencies: uncolored shared parts were viewed more often and thus may have provided more opportunity for participants to imagine their colors. However, part performance was matched across feature types by the end of learning (when the color bias between shared and unique features was at its strongest), suggesting matched engagement with the two feature types.

Curiously, we found marginal evidence for the opposite trend early in learning, with unique features showing more attraction than shared features. This effect is consistent with work showing that attraction early in learning may precede later repulsion (Chanales et al., 2017; Wanjia et al., 2021). However, it is possible that this effect was due to unmatched color exposure during a brief instruction phase we administered at the start of the experiment. Before starting the main experiment, participants completed a short (approximately 4 minutes) introductory exposure phase with the satellites where they clicked each part of each satellite one at a time and viewed the part’s color for 3 seconds. Because shared parts occur more frequently than unique parts, participants had more opportunities to see the color of shared parts (each shared part was seen three times, and each unique part was seen once). To evaluate the potential impact of this exposure phase difference, in our second experiment we matched viewing of shared and unique features during the exposure phase.

### Experiment 2

The purpose of Experiment 2 was to replicate the findings of Experiment 1 while matching the frequency of shared and unique feature colors during the introductory exposure phase. To accomplish the matching, we only showed the color of one shared part per satellite, resulting in each part color being revealed exactly once during this phase. We also made a minor change to the trial randomization procedure for color memory trials (see Methods). Finally, we added a post-learning test phase where we examined how color memory distortions might manifest when making judgments about novel satellites. The experiment protocol was otherwise identical to Experiment 1.

#### Part Learning.

**Figure 3.**
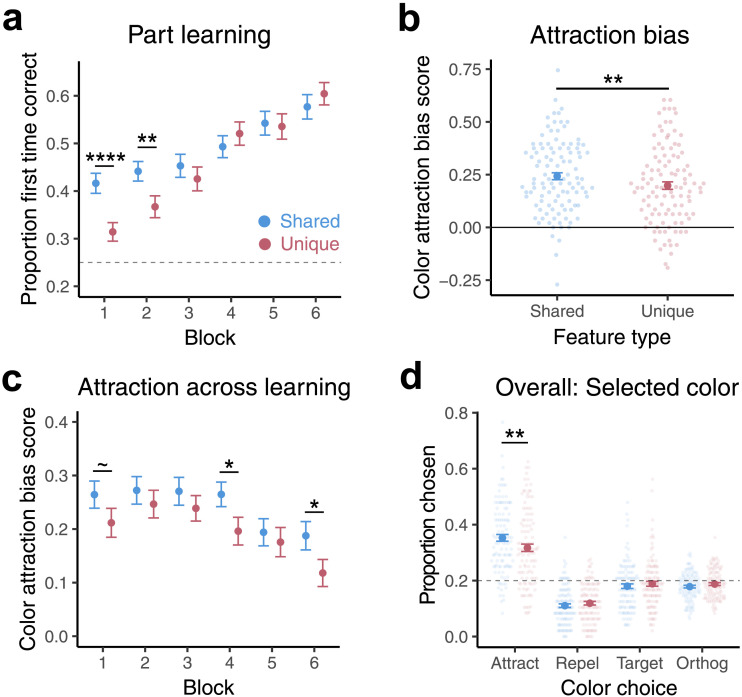
**Part learning and color memory in Experiment 2.** **(a)** The proportion of trials in which the correct satellite part was selected on part trials for shared and unique features across training blocks. **(b)** Overall color attraction bias scores for shared and unique features. **(c)** Color attraction bias scores for shared and unique features across the six blocks. **(d)** The overall proportion of color memory trials in which each color option (Attract, Repel, Target, Orthogonal) was selected for shared and unique features. Significance stars reflect uncorrected *p* values.

Part learning performance closely replicated the results of Experiment 1. There was a main effect of block, with part accuracy improving across the six blocks of learning (*B* = .21, *SE* = .01, *z* = 16.76, *p* < .0001, Figure 3a). Again, while participants had overall better accuracy for shared than unique parts (*B* = −.07, *SE* = .02, *z* = −3.10, *p* = .002), an interaction (*B* = .06, *SE* = .01, *z* = 4.64, *p* < .0001) revealed that this difference disappeared with learning (post-hoc *t* tests: Block 1: *p* < .001, FDR corrected *p* < .001; Block 2: *p* = .003, FDR corrected *p* = .008; Block 3–6: *p*’s > .24).

#### Color Memory.

Unlike Experiment 1, where differences in color memory emerged in an interaction with block, here participants misremembered shared features as more similar to their category center than unique features overall, across blocks. This was evident in both average color attraction bias scores (*t*(108) = 3.00, *p* = .003; Figure 3b) and in the mixed effects model as a main effect of feature type (*B* = −.02, *SE* = .01, df = 10305.1, *t* = −3.69, *p* < .001) and lack of interaction with block (*p* = .68). There was also a main effect of block, with attraction decreasing across learning (*B* = −0.02, df = 10305, *t* = −5.46, *p* < .0001). For comparison to Experiment 1, exploratory slope analyses showed that both shared (*B* = −0.02, *SE* = 0.01, df = 10305, *t* = −3.58, *p* = .0004) and unique feature (*B* = −0.02, *SE* = .01, df = 10305, *t* = −4.15, *p* < .0001) attraction significantly decreased across learning. Attraction scores were significantly above zero overall (*t*(108) = 14.32, *p* < .0001). Exploratory post-hoc *t* tests on attraction bias scores in each block (Figure 3c) showed marginal evidence for greater attraction of shared than unique features in certain blocks (Block 1: *t*(108) = 1.73, *p* = .09, FDR corrected *p* = .17; Block 4: *t*(108) = 2.54, *p* = .01, FDR corrected *p* = .07; Block 6: *t*(108) = 2.28, *p* = .02, FDR corrected *p* = .07; other blocks *p*’s > .26). As in Experiment 1, a model predicting color accuracy on each trial revealed that color accuracy improved overall over time (block main effect: *B* = .09, *SE* = .02, *z* = 6.04, *p* < .0001), with no difference in color accuracy for shared and unique features (feature type main effect: *p* = .33). In this experiment there was a marginal interaction with block (*B* = .03, *SE* = .02, *z* = 1.75, *p* = .08), in the direction of unique features becoming more accurate than shared.

We again assessed which foils participants were selecting on color memory trials. In this case we collapsed across blocks, given the finding of an overall effect of feature type across blocks. Participants again did not differ in how often they chose the target color for shared and unique features (*p* = .30), but they selected the attract color foil more often for shared than unique features (*t*(108) = 3.07, *p* = .003; Figure 3d). No other reliable differences between feature types were observed in foil choices (*p*’s > .12). To directly compare the results across experiments, we also examined the pattern of color selection within the final block of learning. Consistent with Experiment 1, in the last block of learning participants chose the repel foil more often (*p* = .04) and attract foil marginally less often (*p* = .08) for unique than shared features. Again, there was no correlation across subjects between part performance and color attraction biases for either feature type (*p*’s > .40).

As in the last experiment, there was an interaction between color arrangement and feature type (*p* < .0001). But again, the effect of feature type held when color arrangement was added as an interaction term in this model (*p* = .004) and also in a version of the model that added the 16 color coordinates as a factor (*p* = .004).

#### Post-Learning Test and Novel Satellites.

**Figure 4.**
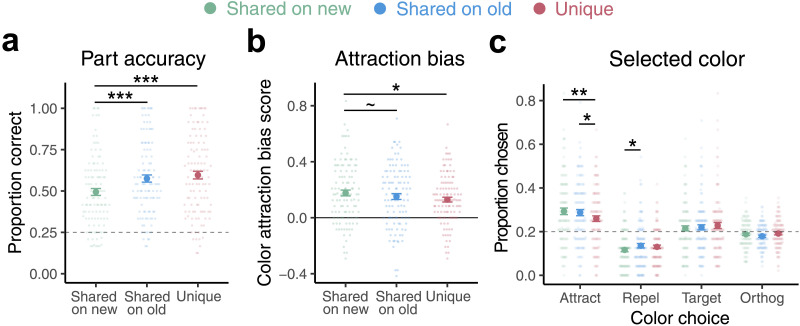
**Post-learning test on trained and novel satellites.** **(a)** The proportion of part trials in which the correct satellite part was selected in the post-learning test phase for shared features on novel satellites (green), shared features on trained satellites (blue), and unique features on trained satellites (red). **(b)** Color attraction bias scores for each feature type. **(c)** The proportion of color memory trials in which each color option was selected for each feature type.

In the post-learning test phase, we evaluated part and color memory for novel as well as trained satellites. A one-way ANOVA revealed differences in part accuracy for unique features of trained satellites, shared features of trained satellites, and shared features of novel satellites (*F*(2, 324) = 6.06, *p* = .003, Figure 4a). Though participants were well above chance for all three feature types (*p*’s < .0001), pairwise *t* tests showed that participants performed equally well at inferring missing shared and unique parts of trained satellites (*p* = .21), but were worse at inferring the missing shared feature of novel satellites (*p*’s < .001). This poorer performance suggests that participants treated previously-seen shared features appearing on novel satellites differently.

We then explored how participants inferred the color of shared features when presented in the context of a novel satellite (Figure 4b). A one-way ANOVA on color attraction scores with the three feature types (shared on novel satellites, shared on trained satellites, unique on trained satellites) was not significant (*p* = .24). Pairwise *t* tests, however, did provide some evidence that these feature types differed in terms of attraction scores. The attraction bias for shared features on novel versus trained satellites was marginally larger (*t*(108) = 1.75, *p* = .08). Attraction for shared features on novel satellites was significantly stronger than for unique features (*t*(108) = 2.54, *p* = .01), whereas there was no reliable difference between shared and unique features of trained satellites in this phase (*t*(108) = 1.12, *p* = .27). There was no correlation in this phase between part accuracy and attraction bias for any of the three feature types (*p*’s > .47).

We also examined which foils participants selected on color trials in this phase (Figure 4c). Relative to unique features, participants were more likely to choose attract foils for shared features on both trained (*t*(108) = 1.98, *p* = .05) and novel satellites (*t*(108) = 2.44, *p* = .02). Participants were more likely to choose repel foils for shared features on trained relative to novel satellites (*t*(108) = −2.10, *p* = .04). No other foil selection differences between feature types were observed (*p*’s > .16). Again, there were no significant pairwise differences across feature types in the tendency to choose the correct color (target) or orthogonal side foils (*p*’s > .10). Together, these data suggest that attraction biases for shared features persisted in the context of novel satellites.

Experiment 2 was a well-powered replication of Experiment 1 where we also matched exposure to part colors in the short initial exposure phase. We again found systematic differences in how the colors of shared and unique parts were misremembered, with shared features showing more attraction to the category center than unique features. Experiment 1 showed this effect emerging with learning, whereas in Experiment 2 it was more evident throughout. We did not replicate the curious effect in Experiment 1 where at first unique features showed marginally more attraction than shared, suggesting that the higher frequency of shared features in the initial phase in Experiment 1 may have made them less prone to attraction early on. Learning the category structure then eventually overrode this initial difference in Experiment 1. Completely balancing exposure frequency in Experiment 2 may have allowed the effect of category structure to emerge earlier. In a post-learning test phase we found that the attraction bias seen for shared features persisted, and was perhaps even stronger, for shared features of novel satellites, consistent with the idea that generalization benefits from a strong representation of the central tendency of the category.

### Neural Network Simulations

**Figure 5.**
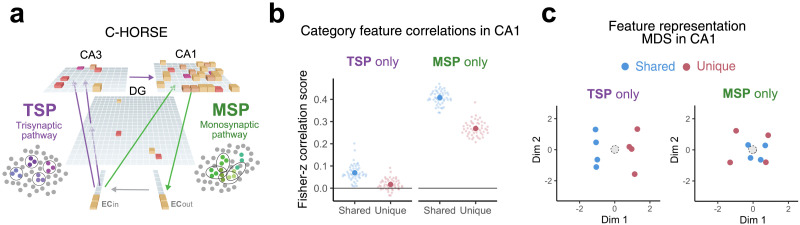
**Feature representations in C-HORSE.** **(a)** Architecture of C-HORSE, a neural network model which instantiates the two main pathways of the hippocampus, the trisynaptic pathway (TSP) and the monosynaptic pathway (MSP). The height and color of the cubes indicate the activity of units at one example cycle of processing. Arrows denote connectivity between model layers, where units in the sending layer have weights connecting to units in the receiving layer. The TSP, with connections shown in purple, tends to keep representations separate, whereas the MSP, shown in green, promotes more overlap. **(b)** Feature-level representational similarity analysis of the initial response in CA1 when the model’s TSP was lesioned (MSP only) or MSP was lesioned (TSP only). Fisher-Z correlation scores were calculated by computing a Pearson correlation for each feature to all other features of the same category. These correlation values were then averaged and Fisher Z-transformed for each model initialization. A higher score indicates that features from the same category were represented more similarly to one another (i.e., more integrated). Small dots represent data from individual model initializations. **(c)** Multidimensional scaling (MDS) plot of feature representations in CA1. Each dot reflects one feature, with the distance between dots corresponding to the distance between those feature representations, averaged across all runs of the model and across the two categories. Gray dashed circles depict the category center (the average coordinate across all features from the same category).

Experiments 1 and 2 leveraged distortions in color memory as a behavioral index for how shared and unique features are represented. We next aimed to simulate these findings in a model where we have direct access to underlying representational structure. There is growing evidence that the hippocampus plays an important role in rapid category learning (Kim et al., 2018; Mack et al., 2018), and we recently provided an account of its specific contributions in a neural network model instantiating biological details of the hippocampus (Sučević & Schapiro, 2023; Figure 5a). The model, C-HORSE, puts forward the theory that the trisynaptic pathway (TSP) is responsible for remembering individual episodes without interference while the monosynaptic pathway (MSP) to CA1 specializes in extracting statistics across these episodes (Schapiro, Turk-Browne, et al., 2017). In the category learning domain, the monosynaptic pathway’s ability to extract statistics leads to effective learning of category structure across exemplars (Sučević & Schapiro, 2023). Consistent with this account, we had found in an fMRI study using the satellite stimuli that satellites from the same category were represented more similarly in CA1 (Schapiro et al., 2018). We trained C-HORSE on the same categories as human participants and examined its learned representations for individual features, evaluating how shared and unique features came to be represented in the two pathways.

To assess model accuracy in feature learning, we analyzed test trials in which full satellite patterns were presented and measured reproduction of the satellite pattern by computing the proportion of correct units in EC_out activated above 0.5. Across training, the model improved in its ability to accurately reconstruct the satellites, achieving 95% accuracy after 6 blocks of training (Supplementary Figure 2). To probe feature representations, we conducted a representational similarity analysis for layer CA1 by correlating the initial response patterns evoked by presentation of each individual satellite feature. We computed the average Fisher Z-transformed correlations between the representations of each feature with other features within the same category. To assess the contribution of each of the two model pathways, we performed this analysis in model versions where each pathway was lesioned, leaving only the MSP or TSP to drive CA1 representations (Figure 5b). Shared features were reliably more integrated than unique features in both lesion variants (MSP only: *t*(49) = 29.33, *p* < .001; TSP only: *t*(49) = 11.05, *p* < .001), but the effect was much stronger in the MSP-driven version (two-way ANOVA interaction between pathway lesion and feature type: *F*(1, 196) = 92.68, *p* < .0001). Consistent with its role in encoding separate representations of individual episodes and the details of individual exemplars (Schapiro, Turk-Browne, et al., 2017; Sučević & Schapiro, 2023), the TSP acted to separate the feature representations regardless of their role in the category, resulting in low correlations for both feature types.

We visualized these feature representations in 2D space with multidimensional scaling (MDS) to illustrate the different feature similarity structures across the pathways and to more directly relate the high dimensional model representations to the behavioral 2D color space. We found that in the MSP-only version of the model, shared features clustered closer to the category center, while unique features remained more peripheral, whereas in the TSP-only version, shared and unique features behaved much more similarly to one another and scattered at similar distances to the category center (Figure 5c).

Overall, as in the human data, the model represented shared features as closer to the category center than unique features. The model suggests that the MSP of the hippocampus could play a particularly important role in generating this effect, as this pathway exhibited especially strong sensitivity to the differences between feature types.

## DISCUSSION

There are general patterns that connect our experiences and details that distinguish them. We found that these different forms of information are represented differently in memory. When learning about categories of novel objects, participants misremembered the color of the object parts differently depending on whether they were shared with other exemplars in the category or were unique to a particular exemplar. Relative to unique features, participants remembered shared features as more similar to the average color of features from that category, suggesting more integration for shared than unique features. This effect emerged as learning progressed in Experiment 1 and was observed overall in Experiment 2. The attraction bias persisted into a post-training test in Experiment 2 when indicating the color for shared features of novel satellites. Simulations with C-HORSE, a neural network model of the hippocampus (Schapiro, Turk-Browne, et al., 2017; Sučević & Schapiro, 2023), recapitulated these results, demonstrating that a simple network rapidly trained to reconstruct the individual satellites accounts for these feature representation patterns. Together, the findings reveal that feature representations are rapidly shaped by the roles they play in a category.

Our work builds on a growing literature demonstrating the utility of continuous memory measures, such as color and location, as a window into how memories are organized (Antony et al., 2022; Berens et al., 2020; Chanales et al., 2021; Richards et al., 2014; Tompary & Thompson-Schill, 2021; Zeng et al., 2021; Zhao et al., 2021). These studies often report attraction biases where participants misremember an item’s associated color or location as more similar to other studied items, suggesting integrated memory representations. We also found evidence for such attraction, with participants misremembering the color of satellite parts as more similar to the average color of their category. We show that the extent of this bias is dependent on the kind of information tested, with shared features showing more susceptibility to attraction than unique ones. This was not explained by more accurate memory for the color of unique features, suggesting representational warping in both cases.

We show that category-based memory distortions manifest at the feature level—different features within the same object can be remembered differently depending on their roles in a category. This is consistent with work emphasizing the importance of feature structure (i.e., how features co-vary within a category) in category learning and representation (e.g., McRae et al., 1997, 1999; Solomon & Schapiro, 2024). Our work also supports the view that objects are not necessarily holistic, bound units (Olson & Jiang, 2002) and that different object features can undergo different courses of forgetting and consolidation (Andermane et al., 2023; Brady et al., 2013; Siefert et al., 2024; Utochkin & Brady, 2020). We extend this literature by showing how discrete features that vary in their typicality within a category exhibit varying susceptibility to memory distortions. A limitation of the present study is that the feature inference learning task we employ explicitly directs attention to individual features, potentially leading participants to treat object features as more separable than they otherwise would. Indeed, mode of learning—inferring missing features versus classifying objects into categories versus passive observation—is known to shape how people learn and represent categories (Anderson et al., 2002; Ashby et al., 2002; Chin-Parker & Ross, 2002; Hoffman & Rehder, 2010; Levering & Kurtz, 2015; Minda & Ross, 2004; Yamauchi & Markman, 1998), and real-world learning may often involve more passive forms of exposure. It will be interesting for future work to assess whether and how feature memory distortion of the kind we report is modulated by learning mode. Future work will also be needed to investigate how these memory distortions generalize to real-world objects and more complex stimuli (Dubova & Goldstone, 2021; Son et al., 2022). This study focuses on one particular kind of structure (discrete features that are either mostly shared or entirely unique), which describes some kinds of ecological categories well (e.g., mammals have fur, but bats are the only mammals that can fly), but other kinds of real-world categories have more mixed and continuous features.

Related feature-level distortions have also been documented in the literature on categorical perception (Dubova & Goldstone, 2021; Folstein et al., 2015; Goldstone, 1994; Goldstone & Hendrickson, 2010), where features near a category boundary are perceived (or remembered; Firestone & Scholl, 2016) as more distinct. In these studies, there are typically continuous feature dimensions that span categories, with a boundary distinguishing them. The present study differs from categorical perception by investigating how discrete features varying in typicality within an individual category are subject to memory distortions. We also demonstrate memory distortions for an attribute (color) that does not itself carry information about feature roles (whether a feature is unique or shared) in the category. Despite these differences, the present work builds on the categorical perception literature in illustrating how category learning systematically shapes how we perceive and remember.

Our findings also show how newly learned categories can very rapidly drive distortions in memory, consistent with reported mnemonic and perceptual distortions in other novel category learning studies (Dubova & Goldstone, 2021; Goldstone, 1994; Livingston et al., 1998; Son et al., 2024). Other research has shown how pre-existing knowledge of well-established semantic concepts, such as categories of real-world animals or objects (Brady et al., 2018; Tompary & Thompson-Schill, 2021; Tompary et al., 2023), can distort newly encoded memories in a way that is highly sensitive to category structure. In Tompary and Thompson-Schill (2021), participants memorized the locations of naturalistic images (e.g., birds) clustered in a 2D spatial array. They found that memory of the item locations (individual birds) was biased towards the average location of their category in space. Atypical category members (e.g., penguin), however, were less prone to this attraction bias, analogous to the behavior we observed for unique features. There is evidence that neocortical semantic representations drive these category-based distortions of episodic encoding (Tompary et al., 2023). Because memory distortions systematically change with time and consolidation (Zeng et al., 2021), it will be useful for future work to test whether different memory mechanisms and brain regions drive distortions during initial category learning (of the kind we study here) versus for well-established semantic knowledge.

A topic of recent interest in the memory literature has been the conditions under which memory traces integrate versus separate (Bein et al., 2023; Brunec et al., 2020; Chanales et al., 2021; Ritvo et al., 2019; Schlichting et al., 2015). We show that the role a feature plays within a category may be an important determinant of this. However, while shared features showed more attraction than unique ones, we still saw strong overall attraction biases for both feature types in our paradigm. In other words, we never observed true repulsion, where related information is represented or remembered as more distinct than unrelated information (Chanales et al., 2017, 2021; Chunharas et al., 2018; Drascher & Kuhl, 2022; Favila et al., 2016; Wanjia et al., 2021; Zhao et al., 2021). For example, Drascher and Kuhl (2022) found that participants misremembered pairs of faces as less similar to one another (repulsion) along the facial feature dimension most diagnostic for differentiating the two faces. This finding is consonant with the findings we report here for unique features, where information that is diagnostic of an item showed reduced attraction (though not repulsion). One possibility is that true repulsion for unique features would have emerged if the training period in our study was extended longer, as both experiments found decreasing attraction for unique features over learning. Indeed, repulsion emerges after extensive amounts of training often resulting in near-ceiling performance (Chanales et al., 2017; Wanjia et al., 2021). Another possibility is that repulsion emerges only when there is a strong demand for interference reduction (e.g., when the task requires disambiguating extremely similar stimuli; Chanales et al., 2021).

We provide a demonstration of how our shared and unique feature distortion effects could arise by simulating our paradigm using a neural network model of the hippocampus (Schapiro, Turk-Browne, et al., 2017), where we have direct access to the structure of learned representations. We used this model because of evidence of hippocampal involvement in both the satellite learning paradigm (Schapiro et al., 2018) and in rapid novel category learning more generally (Kim et al., 2018; Mack et al., 2018). We found that the MSP, implicated in statistical learning and category learning (Schapiro, Turk-Browne, et al., 2017; Sučević & Schapiro, 2023), represented shared features (relative to unique features) more similarly to other features within the same category, analogous to the attraction bias in humans. The TSP, in contrast, well-known for its pattern separation and episodic memory functions, tended to separate all feature representations, with less sensitivity to their role in the category. The model was trained to reconstruct each satellite’s configuration of features. Because shared features are predictive of the presence of other shared features in a satellite, the distributed representations of the MSP tend to pull the internal representation of shared features toward one another. This pathway of the model behaves as a rapid-learning but otherwise standard three layer neural network model employing distributed representations in its hidden layer (Hinton, 1984), and so we expect that many neural network models employing distributed representations would exhibit the same phenomenon. Indeed, there is a long history of showing how distributed representations come to reflect both the general and specific features of their training environment (McClelland & Rumelhart, 1985; Rogers & McClelland, 2004; Saxe et al., 2019), though this is typically explored at the level of items, as opposed to features within an item, and with more extensive training. Our prior category learning simulations with this model and paradigm demonstrated that the TSP was needed for accurate output reconstruction of unique features, whereas the MSP was sufficient for reconstruction of shared features (Sučević & Schapiro, 2023). This means that the representation of unique features we observe in the MSP does not correspond to a high fidelity trace of those features that would support their retrieval but instead reflects their more general role in the category. Overall, the model shows how a simple, rapidly learned distributed representation, as implemented in the MSP of this hippocampus model, can exhibit the distortions observed in our behavioral experiments. Future neuroimaging studies can test these ideas about hippocampal contributions as well as investigate interactions with other relevant brain areas (Clarke et al., 2016; Zhao et al., 2021).

Taken together, we show that memory systems come to mirror the structure of their environment, with commonalities represented closer together and unique details kept relatively separate. This learning of structure rapidly manifests in behavior as different memory distortions for individual features. Memory systems thus seem to solve the problem of learning both general and specific information by representing features differently as a function of their properties.

## ACKNOWLEDGMENTS

We thank Dhairyya Singh for consultation on the model simulations and Timothy Brady for helpful discussions on the color memory paradigm.

## FUNDING INFORMATION

This work was supported by National Institutes of Health grant R01 MH129436 to A.C.S. and Natural Sciences and Engineering Research of Canada postgraduate scholarship to M.C.T.

## AUTHOR CONTRIBUTIONS

M.C.T.: Conceptualization; Data curation; Formal analysis; Funding acquisition; Investigation; Methodology; Software; Visualization; Writing – original draft; Writing – review & editing. C.V.D.: Data curation; Formal analysis; Investigation; Methodology; Software; Visualization; Writing – original draft; Writing – review & editing. A.C.S.: Conceptualization; Funding acquisition; Methodology; Project administration; Supervision; Writing – review & editing.

## DATA AVAILABILITY STATEMENT

Stimuli, data, and analysis code are available at github.com/schapirolab/color-cat.

## Supplementary Material


