# Supplementary material for: Object Feature Memory Is Distorted by Category Structure
Source: Open Mind (Camb). 2024 Nov 22;8:1348–68. doi: 10.1162/opmi_a_00170 (PMC11627532; doi:10.1162/opmi_a_00170)
Supplement: Supplementary file 1 [file opmi-08-1348-s001.pdf]

Supplementary Material

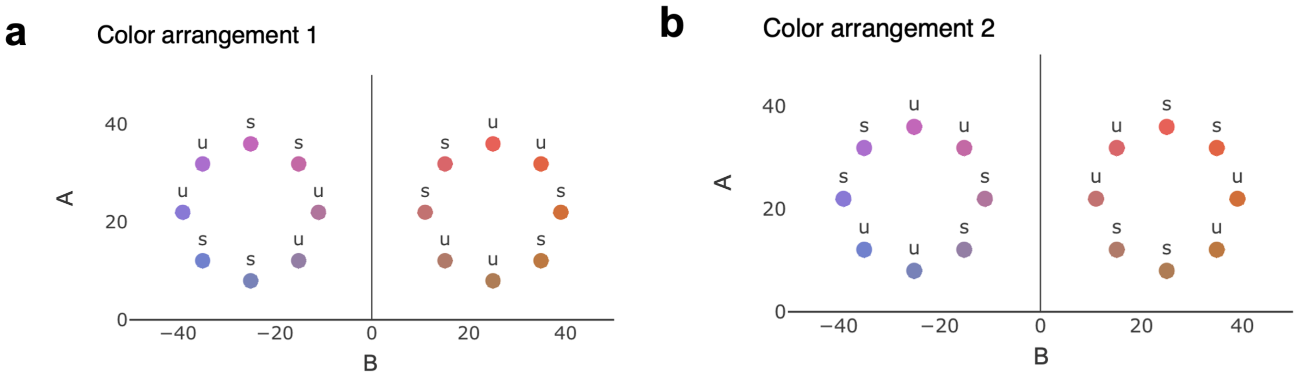

Supplementary Figure 1. The two feature color arrangements. The only difference between the two randomly assigned color arrangements were that shared and unique features were swapped. s = shared feature, u = unique feature. The y-axis is the A coordinate and the x-axis is the B coordinate in CIELAB color space. L was fixed at 60 for both arrangements. Colored dots depict actual color at that coordinate.

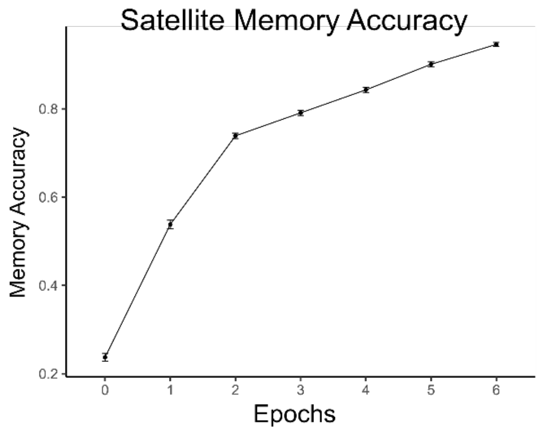

Supplementary Figure 2. Satellite reconstruction accuracy in the neural network model. To assess model accuracy in each epoch, we analyzed the test trials in which full satellite patterns were presented. We assessed reproduction of the satellite pattern by computing the proportion of correct units in EC_out activated above 0.5. Across training, the model improved in its ability to accurately reconstruct the satellites.

			Category 1													Category 2												
**Type**	**Cat**	**ID**	**Cl1**	**Cd1**	**Cd2**	**Cd3**	**Cd4**	**S1**	**S2**	**S3**	**S4**	**U1**	**U2**	**U3**	**U4**	**Cl2**	**Cd5**	**Cd6**	**Cd7**	**Cd8**	**S5**	**S6**	**S7**	**S8**	**U5**	**U6**	**U7**	**U8**
Item	1	I1	1	1	0	0	0	0	1	1	1	1	0	0	0	0	0	0	0	0	0	0	0	0	0	0	0	0
Item	1	I2	1	0	1	0	0	1	0	1	1	0	1	0	0	0	0	0	0	0	0	0	0	0	0	0	0	0
Item	1	I3	1	0	0	1	0	1	1	0	1	0	0	1	0	0	0	0	0	0	0	0	0	0	0	0	0	0
Item	1	I4	1	0	0	0	1	1	1	1	0	0	0	0	1	0	0	0	0	0	0	0	0	0	0	0	0	0
Item	2	I5	0	0	0	0	0	0	0	0	0	0	0	0	0	1	1	0	0	0	0	1	1	1	1	0	0	0
Item	2	I6	0	0	0	0	0	0	0	0	0	0	0	0	0	1	0	1	0	0	1	0	1	1	0	1	0	0
Item	2	I7	0	0	0	0	0	0	0	0	0	0	0	0	0	1	0	0	1	0	1	1	0	1	0	0	1	0
Item	2	I8	0	0	0	0	0	0	0	0	0	0	0	0	0	1	0	0	0	1	1	1	1	0	0	0	0	1
Feature	1	S1	0	0	0	0	0	1	0	0	0	0	0	0	0	0	0	0	0	0	0	0	0	0	0	0	0	0
Feature	1	S2	0	0	0	0	0	0	1	0	0	0	0	0	0	0	0	0	0	0	0	0	0	0	0	0	0	0
Feature	1	S3	0	0	0	0	0	0	0	1	0	0	0	0	0	0	0	0	0	0	0	0	0	0	0	0	0	0
Feature	1	S4	0	0	0	0	0	0	0	0	1	0	0	0	0	0	0	0	0	0	0	0	0	0	0	0	0	0
Feature	1	U1	0	0	0	0	0	0	0	0	0	1	0	0	0	0	0	0	0	0	0	0	0	0	0	0	0	0
Feature	1	U2	0	0	0	0	0	0	0	0	0	0	1	0	0	0	0	0	0	0	0	0	0	0	0	0	0	0
Feature	1	U3	0	0	0	0	0	0	0	0	0	0	0	1	0	0	0	0	0	0	0	0	0	0	0	0	0	0
Feature	1	U4	0	0	0	0	0	0	0	0	0	0	0	0	1	0	0	0	0	0	0	0	0	0	0	0	0	0
Feature	2	S5	0	0	0	0	0	0	0	0	0	0	0	0	0	0	0	0	0	0	1	0	0	0	0	0	0	0
Feature	2	S6	0	0	0	0	0	0	0	0	0	0	0	0	0	0	0	0	0	0	0	1	0	0	0	0	0	0
Feature	2	S7	0	0	0	0	0	0	0	0	0	0	0	0	0	0	0	0	0	0	0	0	1	0	0	0	0	0
Feature	2	S8	0	0	0	0	0	0	0	0	0	0	0	0	0	0	0	0	0	0	0	0	0	1	0	0	0	0
Feature	2	U5	0	0	0	0	0	0	0	0	0	0	0	0	0	0	0	0	0	0	0	0	0	0	1	0	0	0
Feature	2	U6	0	0	0	0	0	0	0	0	0	0	0	0	0	0	0	0	0	0	0	0	0	0	0	1	0	0
Feature	2	U7	0	0	0	0	0	0	0	0	0	0	0	0	0	0	0	0	0	0	0	0	0	0	0	0	1	0
Feature	2	U8	0	0	0	0	0	0	0	0	0	0	0	0	0	0	0	0	0	0	0	0	0	0	0	0	0	1

Supplementary Table 1. Model input patterns for training and testing. The eight satellite items (ID = I1-I8) in the top portion of the table (Type = Item) were used as input for model training. Each satellite had a class name (Cl), a unique code name (Cd), three shared features (SX), and one unique feature (UX). This same input was used for the reconstruction test. Feature-level representational similarity analyses were conducted during testing with the inputs on the lower half of the table (Type = Feature). Cat = category, Cl = class name, Cd = code name, S = shared, U = unique.
